# Effects of direct-fed microbial supplementation on feedlot lamb growth performance, serum biochemical markers, fecal consistency, and fecal microbiota

**DOI:** 10.1093/tas/txag085

**Published:** 2026-06-19

**Authors:** Kristen E Kahler, Kelsey L Bentley, Payton L Dahmer

**Affiliations:** Department of Animal Sciences and Industry, Kansas State University, Manhattan, KS, United States

**Keywords:** direct-fed microbial, feedlot lambs, growth, health

## Abstract

Dietary, environmental, and physiological changes can challenge lamb health and performance during the feedlot transition. This study evaluated supplementation with a liquid direct-fed microbial (DFM; RSG Prime by BIO S.I.) on growth, fecal consistency, blood parameters, and fecal microbial populations in feedlot lambs fed a high-concentrate diet. Seventy-two Dorper × Katahdin lambs (60 d of age, initially 26.73 ± 0.35 kg BW) were fed for 63 d at Kansas State University. Lambs were randomly assigned by initial body weight to pens (3 lambs/pen) and allotted to one of two treatments during the treatment period (d 0–63), with 12 replications per treatment. Treatments were (1) control (CON, no DFM) or (2) DFM supplementation (15 mL/head, orally once daily at 08:00). The DFM contained *Bacillus licheniformis* (10 million CFU/mL), *Bacillus subtilis* (3.5 million CFU/mL), *Lactobacillus acidophilus* (3 million CFU/mL), and *Bacillus pumilus* (2 million CFU/mL). Lambs and feeders were weighed weekly to determine average daily gain (ADG), feed intake (ADFI), and gain-to-feed ratio (G: F). Feces were scored weekly (d 0–42). Blood was collected on d 0 and 21 from one lamb/pen, and fecal samples were collected for microbial analysis. No differences (*P* > 0.05) were observed for ADG, ADFI, or G: F. Fecal scores were unaffected by treatment (*P* > 0.05) but improved over time (*P* < 0.0001). No treatment × day interactions were detected for blood variables. Serum calcium tended to be lower (*P* = 0.06) in lambs supplemented with a DFM. Albumin and anion gap decreased significantly from d 0 to 21 (*P* ≤ 0.01), while glucose, urea nitrogen, globulin, magnesium, and bicarbonate increased (*P* ≤ 0.03). Calcium and potassium tended to be greater in the control group (*P* ≤ 0.10). Relative abundance of several genera in the feces showed a significant treatment effect (*P* ≤ 0.05) There were statistically significant shifts *(P* ≤ 0.05) in fecal microbial populations from d 0 to 21. *Opitutae, Bifidobacteriales, Treponema*, and *Bifidobacterium* showed treatment × day interactions (*P* ≤ 0.05). In summary, DFM supplementation did not significantly alter growth performance or fecal consistency in feedlot lambs. Lambs supplemented with a DFM had alterations in serum chemistry and changes in fecal microbial populations.

## Teaser

Probiotic supplementation did not improve lamb growth during the treatment period. However, blood parameters and fecal microbiota showed positive shifts, suggesting lasting benefits from early gut health support.

## Introduction

The transition to a feedlot environment presents significant physiological and nutritional challenges for lambs. During this period, lambs often experience stress caused by environmental and dietary changes. Increased exposure to pathogens can weaken the immune system, and when combined with dietary shifts, this can lead to a decline in lamb growth performance. Typically, lambs are moved from a low-concentrate diet alongside their dams to a high-concentrate feedlot ration, a shift that has been associated with an increased risk of ruminal acidosis due to the rapid introduction of dietary starch (Braun et al. 1992). The ruminal microbiome is heavily affected by feed composition and digestion. Microbial shifts occur due to changes in the contents of the rumen, with high forage diets increasing bacterial and fungal populations (Neves et al. 2025).

When high amounts of nonstructural carbohydrates are ingested, the imbalance of microbial populations causes the ruminal pH to drop below 5.4, potentially resulting in epithelial cell damage or death (Brown et al. 2000). The absorption of volatile fatty acids is lowered, causing them to accumulate in the rumen, while the influx of bicarbonate from the bloodstream is reduced. This, in conjunction with damaged ruminal epithelium, allows lipopolysaccharides to translocate into the bloodstream, triggering systemic inflammation and metabolic inefficiency (Minuti et al. 2014). Clinical signs of ruminal acidosis include teeth grinding, anorexia, diarrhea, ruminal stasis, and elevated levels of lactic acid that disrupt metabolic processes (Streitenberger et al. 2025). In severe cases, treatment may require the transfer of rumen fluid to reestablish microbial populations and restore pH balance (Braun et al. 1992). Acidosis has been shown to reduce feed intake and efficiency, causing economic problems for producers. While antimicrobials have traditionally been used to mitigate acidosis (Dirksen and Khamis 1974; Reis et al. 2018), growing concerns over antibiotic resistance and residues in meat products have spurred interest in alternative strategies (Menkem et al. 2019; Getahun et al. 2023). This consumer and regulatory pressure to limit the use of antibiotics has pushed the industry to explore alternatives.

Common treatment alternatives to antibiotics include buffers such as sodium bicarbonate and magnesium oxide, pre and probiotics, and ionophores (Enemark 2008). Buffers create efficiency in rumen digestion by maintaining a balanced pH, keeping acidity and alkalinity in check, which allows microbiota to flourish (Kumar et al. 2024). Direct-fed microbials (DFMs) include viable bacteria that can cause a positive shift in the rumen microbiome by increasing lactic acid-utilizing bacteria. DFMs combine lactate-producing and lactate-utilizing bacteria to reduce the severity of acute acidosis in ruminants. Microorganisms can secrete antimicrobial metabolites, compete with pathogenic microbes, and degrade environmental toxins–collectively enhancing host health and metabolic efficiency (Brashears et al. 2003; Ramirez-Olea et al. 2022). DFMs have also been shown to improve gastrointestinal tract morphology under acidotic stress (Dick et al. 2013).

On the other hand, ionophores improve efficiency and rate of gain by manipulating the transport of ions across membranes, increasing gram-negative bacteria (Spears 1990). By altering the concentrations of fermentation end products and bacterial populations, ionophores and DFMs have been shown to improve feed conversion efficiency (Seo et al. 2010; Ban and Guan 2021; Mombach et al. 2021).

Ionophores and DFMs have demonstrated potential in stabilizing ruminal pH and preventing acidosis (Yoon and Stern 1995). Supplementation with DFMs has been associated with improvements in animal performance (Ban and Guan 2021; Guimaraes et al. 2024), behavior associated with increased feed intake (Lopez et al. 2024), and gut morphology (Dick et al. 2013). The live microbial cultures in DFMs enhance gut microbial balance, reduce disease susceptibility, and support immune function (Guimaraes et al. 2023). This microbial modulation also optimizes fermentation profiles, including reductions in the acetate-to-propionate ratio and improved ruminal ammonia utilization (Dias et al. 2022). These benefits contribute to overall efficiency, increasing G: F.

Notably, spore-forming *Bacillus* species such as *B. licheniformis* and *B. subtilis*, commonly used in DFMs, enhance rumen functionality by promoting the growth of proteolytic bacteria (Green et al. 1999). These organisms produce amylase and cellulase enzymes that aid in the digestion and assimilation of dietary starch, aiding metabolism when a high-starch diet is being fed (Deng et al. 2018). Their colonization in the rumen positively influences nitrogen metabolism and gut function (Sun et al. 2013).

The present study hypothesized that DFM supplementation could support lambs during the challenging transition to a high-concentrate feedlot diet by enhancing performance and feed efficiency. Specifically, *Bacillus* species in the DFM were expected to secrete lactase, increasing ruminal propionate concentrations through lactate utilization (Ramirez-Olea et al. 2022). Previous findings support that DFM formulations containing *Bacillus* spp. improve both growth metrics and health outcomes in lambs (El-Sayed and Mousa 2020). Additionally, supplementation with *Lactobacillus acidophilus* has been shown to reduce *Escherichia coli* shedding in feedlot cattle, indicating broader benefits when feeding this product for animal health and productivity (Krehbiel et al. 2003; Younts-Dahl et al. 2005). The objective of this study was to evaluate the effects of a liquid DFM (RSG Prime by BIO S.I.) on the growth performance, fecal consistency, fecal microbiota, and serum metabolites in feedlot lambs.

## Materials and methods

All experimental procedures adhered to guidelines for the ethical and humane use of animals for research according to the Guide for the Care and Use of Agricultural Animals in Research and Teaching (FASS, 2010) and were approved by the Institutional Animal Care and Use Committee at Kansas State University (IACUC #4953).

### Animals, housing, and experimental design

A total of 72 Dorper X Katahdin wether and ewe lambs were used in a 63-d feeding trial at the Kansas State University Sheep and Meat Goat Center (Manhattan, KS). Lambs were housed in an environmentally controlled facility where temperature, ventilation, and lighting were monitored daily. Lambs were received at approximately 60 d of age and weighed approximately 26.73 ± 0.03 kg initially. Lambs had access to free-choice hay and water upon arrival. After 24 hours of acclimation, all lambs were weighed, tagged, and vaccinated for *Clostridium perfringens* Types C and D and tetanus (BAR-VAC CD/T; Boehringer-Ingelheim). All lambs were FAMACHA scored, and if mucus membranes scored above a 3 out of 5, lambs were dewormed (Valbazen albendazole oral suspension; Zoetis Inc., Parsippany-Troy Hills, NJ). Lambs were then balanced for sex and allotted to pens in a completely randomized design. Lambs were randomly placed into pens (measuring 1.22 m × 0.91 m) with 3 lambs/pen either 2 ewes and 3 wethers or 3 wethers and 2 ewes). Each pen was lined with barriers to prevent nose-to-nose contact of animals during the experiment to limit the risk of cross-contamination between treatments. Each pen was then randomly assigned to one of two treatment groups to provide 12 replications per treatment: (1) Negative control (CON): no DFM supplementation, (2) Direct-fed microbial (DFM): oral supplementation of 15 mL/day of RSG Prime by Bio S.I. (blend of 10 million CFU/mL *Bacillus licheniformis*, 3.5 million CFU/mL *Bacillus subtilis*, 3 million CFU/mL *Lactobacillus acidophilus*, and 2 million CFU/mL *Bacillus pumilus*).

### Diets and experimental timeline

Upon allotment on experiment d -7, lambs were limit-fed for 7 days, and the feed allowance was gradually increased until a target DMI of approximately 3.5% BW was reached. During this time, there was no DFM treatment administered. After the 7-d acclimation period, lambs were gradually transitioned to ad libitum access to a 16% crude protein concentrate diet containing roughage (dehydrated alfalfa) to eliminate the need for supplemental forage (Table 1). At experiment d 0, lambs assigned to the DFM treatment received 15 mL of the DFM via oral drench. This treatment was administered daily until study conclusion at 08:00 each day. The experimental period included a 63-d feeding length, with lambs and feeders weighed weekly to determine average daily gain (ADG), average daily feed intake (ADFI) on an as-fed basis, and feed efficiency (G: F).

**Table 1:** Formulation of standard diet and calculated nutrient composition.^a^

Ingredient	% of Diet
***Corn***	39.71
***Soybean meal, 48% CP***	15.00
***Soybean hulls***	35.70
***Dehydrated alfalfa***	3.00
***Ammonium chloride***	1.00
***Limestone***	1.60
***Sodium chloride***	0.50
***Selenium selenite***	0.001
***Vitamin A 30,000 IU***	0.015
***Vitamin D 30,000 IU***	0.004
***Vitamin E 20,000 IU***	0.001
***Zinc oxide***	0.008
***Dicalcium phosphate***	0.96
***Molasses***	2.50
***Total***	100.00
*** Calculated analysis (as-fed basis)***	
*** Crude protein, %***	16.0
*** Crude fat, %***	2.0
*** Crude fiber, %***	17.0
*** Calcium, minimum, %***	0.9
*** Calcium, maximum, %***	1.3
*** Phosphorus Minimum, %***	0.5

a A standard diet was formulated to meet nutrient requirements for lambs and contained roughage to eliminate feeding of additional forage. The same diet was fed to all animals and nutrient values were calculated from formulated values.

### Fecal consistency and microbiota analysis

Fecal scoring was conducted by two independent, trained scorers weekly from d 0 to d 42 to categorize the consistency of lamb feces per pen. A numerical scale from 1 to 5 was used: 1 being hard pellet-like feces, 2 a firm formed stool, 3 a soft moist stool that retains shape, 4 a soft unformed stool, and 5 a watery liquid stool. On d 0 and 21, fresh fecal samples were collected from one lamb per pen closest to the treatment average BW (*n* = 12 lambs/treatment, 24 lambs total) for fecal microbiota analysis. Fecal samples collected on day 0 were obtained prior to initiation of DFM supplementation and were considered baseline measurements. Consequently, statistical analyses evaluating treatment effects were conducted using post-treatment sampling days. The same lamb was sampled at both time points. Fecal microbiota were evaluated from a single lamb per pen, which may not fully capture within-pen individual variability. However, the selected lamb was intended to be representative of the pen, and this sampling approach allowed evaluation of treatment-associated microbial responses while maintaining consistency across experimental units. Samples were collected into sterile, DNA-free centrifuge tubes and stored a −80° C until shipment to a commercial laboratory (MR DNA, Shallowater, TX) for DNA extraction and taxonomic analysis.

Genomic DNA was isolated from each sample using the PowerSoil DNA Isolation Kit (Qiagen Inc., Valencia, CA). Approximately 250 mg of fecal matter was added to a PowerBead (Qiagen Inc., Valencia, CA) tube and homogenized using the PowerLyzer (Qiagen Inc., Valencia, CA) to induce cell lysis. The purified DNA was then eluted and stored at −20° C until PCR amplification. The 16S universal primers 515F (GTGYCAGCMGCCGCGGTAA) and 806R (GGACTACNVGGGTWTCTAAT) were utilized to amplify the 16S gene of samples on the Illumina NovaSeq (Illumina Inc., San Diego, CA) via the bTEFAPâ DNA analysis service (Dowd et al. 2008a). Each sample underwent a single-step 35 cycle PCR using the Qiagen ALLtaq Plus Master Mix Kit (Qiagen, Valencia, CA) under the following conditions: 95°C for 5 minutes, followed by 30 cycles of 95°C for 30 seconds; 53°C for 40 seconds; and 72°C for 1 minute; after which a final elongation step at 72°C for 10 minutes was performed. Following PCR, all amplicon products from different samples were mixed in equal concentrations and purified using calibrated solid phase reversible immobilization (SPRI) beads. Samples were sequenced utilizing the Illumina NovaSeq (Illumina Inc., San Diego, CA) chemistry following the manufacturer’s protocols.

Data processing was conducted using a proprietary analysis pipeline (MR DNA, Shallowater, TX). Sequences were depleted of primers, short sequences (<150 bp) were removed, and sequences with ambiguous base calls were removed. Sequences were quality filtered using a maximum expected error threshold of 1.0 and dereplicated. The dereplicated or unique sequences were denoised; unique sequences identified with sequencing or PCR point errors were removed, followed by chimera removal, thereby providing a denoised sequence or operational taxonomic unit (OTU). Final OTUs were taxonomically classified using BLASTn (Altschul et al. 1990) against a curated database derived from NCBI (www.ncbi.nlm.nih.gov). Alpha and beta diversity analyses were conducted as previously described by (Dowd et al. 2008b; Edgar 2017) using Qiime v.2 (Boylen et al. 2019). After stringent quality sequence curation, a total of 23,311,504 sequences were identified and utilized for final microbiota analyses. The average reads per sample were 24,336. For alpha and beta diversity analysis, samples were rarefied to 48,000 sequences.

Statistical analysis as well as all alpha and beta diversity analyses were performed using a variety of computer packages including XLstat, NCSS 2007, R, and NCSS 2010. Alpha and beta diversity analysis was conducted using QIIME2 as described previously (Dowd et al. 2008b; Edgar 2010; Cephas et al. 2011; Eren et al. 2011; Bolyen et al. 2018).

### Serum metabolites

On d 0 and 21, whole blood samples were collected from one lamb per pen that was closest to the treatment average BW (*n* = 12 lambs/treatment, 24 lambs total) to analyze serum metabolites. The same lambs were sampled at each time point. Samples were collected from the jugular vein into sterile 10 mL vacuum-sealed tubes (Monoject Blood Collection Tube, Mansfield, MA) using a 22 gauge 1” blood collection needle (Fisher Scientific, Hampton, NH). Samples were immediately placed on ice until transport to the Kansas State University Veterinary Diagnostic Laboratory (Kansas State University, Manhattan, KS) for a complete blood panel, serum chemistry, and hepatic profile analysis via spectrophotometry. Briefly, samples were centrifuged for 5 min at 3000 rpm (Eppendorf North America, Enfield, CT) to separate the serum for analysis. Chemistry assays were then performed utilizing the Cobas c501 (Roche Diagnostics, Indianapolis, IN). Glucose, urea nitrogen, albumin, globulin, total calcium, phosphorus, total magnesium, sodium, potassium, chloride, bicarbonate, anion gap, sodium potassium ratio, aspartate transaminase, plasma pyridoxal 5’-phosphate (P5P), alkaline phosphatase, sorbitol dehydrogenase, and creatine kinase were all analyzed for each sample.

### Statistical analysis

Growth performance and fecal consistency data were analyzed as a completely randomized design with pen as the experimental unit because lambs were group-housed and feed intake was measured at the pen level. Therefore, feed intake and derived performance variables could not be attributed to individual animals. While this approach does not capture individual variation among lambs within a pen, it reflects the unit at which treatments were applied and feed intake was measured. Fecal consistency scores were analyzed using a linear mixed model treating the score as a continuous response variable. Sampling day was included as a repeated measure to account for multiple observations collected over time. For fecal microbiota and serum metabolite data, individual lamb was the experimental unit. Data were analyzed using the GLIMMIX procedure of SAS (v.9.4, SAS Inst., Cary, NC) with the model including treatment, sampling day, and treatment × sampling day interaction as fixed effects. Initial body weight was evaluated as a potential covariate for growth performance variables but was not included in the final model because it was not significant (*p* > 0.10).

Fecal microbiota data were analyzed with individual lamb as the experimental unit to represent one sample selected from each pen on each sampling day (d 0 and d 21). Sampling day was included as a repeated measure to account for multiple observations collected from the same lamb over time. The relative abundance was determined as the total proportion of reads for a sample classified into the specific microbial phyla or family. Samples with a low relative abundance (<0.01%) were excluded from the analysis. Once the relative abundance for each sample was calculated, data were analyzed as a completely randomized design with the model including dietary treatment, sampling day, and their interaction as fixed effects. Data were analyzed as repeated measures given the two sampling time points on d 0 and d 21. Statistical comparisons of observed features and Shannon Diversity indices for each treatment group were conducted using Kruskal-Wallis pairwise comparisons. Beta diversity was analyzed using a weighted UniFrac distance matrix with pairwise analysis of similarities (ANOSIM) used to evaluate differences in microbial communities between groups over time.

Serum metabolite data were log transformed prior to analysis. With individual lamb as the experimental unit, the data were analyzed using the GLIMMIX procedure of SAS (v.9.4, SAS Inst., Cary, NC). The model included the main effects of treatment and sampling day, as well as the treatment × sampling day interaction. The LS Means were utilized to partition treatment differences, and all comparisons included Tukey-Kramer multiple comparison adjustments. Significance was determined at *p* < 0.05 and tendency at 0.05 ≤ *p* < 0.10.

## Results and discussion

### Lamb growth performance and fecal consistency

**Figure 1:**
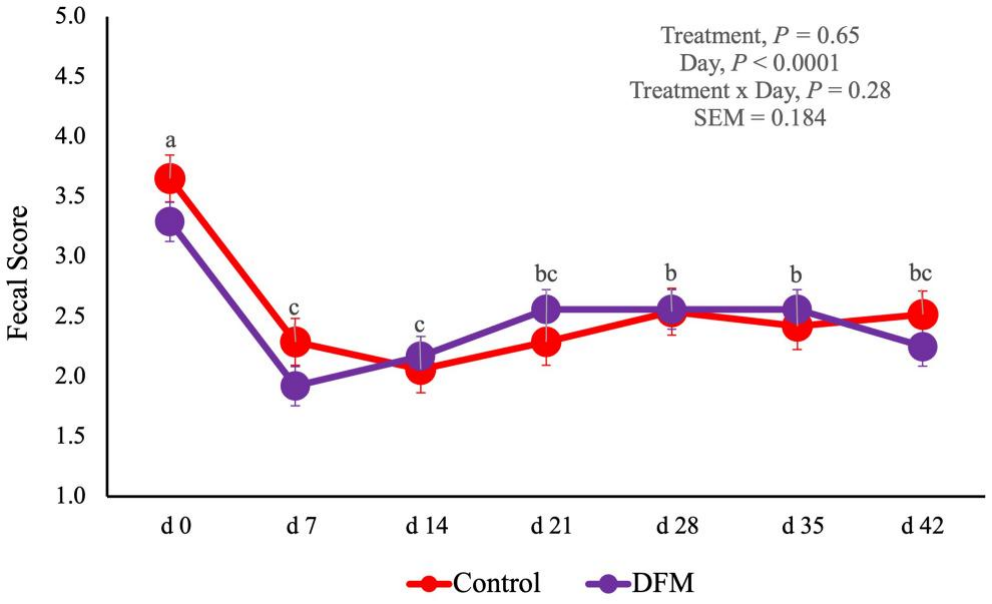
Effect of a liquid direct-fed microbial (DFM) on feedlot lamb fecal score. On days 0, 7, 14, 21, 28, 35, and 42 fecal scoring was conducted by two independent, trained scorers to categorize the consistency of lamb feces per pen. A numerical scale from 1 to 5 was used: 1 being hard pellet-like feces, 2 a firm formed stool, 3 a soft moist stool that retains shape, 4 a soft unformed, and 5 a watery liquid stool. Days without a common superscript differ significantly, *P* < 0.05.

BW and growth performance variables were not affected by treatment throughout the 63-d experimental period (*p* ≥ 0.20; Table 2). There was no evidence of a difference in ADG over the experimental period (*p* = 0.35). Likewise, ADFI (*p* = 0.79) and G: F (*p* = 0.20) were statistically similar across treatments. Collectively, this indicates that supplementation with a DFM did not significantly impact feedlot lamb growth performance compared to a control under the conditions of this trial. Treatment had no effect on fecal scores (*p* > 0.05, Fig. 1), indicating that fecal consistency was not altered by DFM supplementation. Nevertheless, fecal scores improved over time (*p* < 0.0001) in both groups, with a reduction observed from d 0 to d 42. This trend may reflect the natural stabilization of gastrointestinal function as lambs matured and adapted to their diet.

**Table 2:** Effect of a liquid direct-fed microbial (DFM) on feedlot lamb growth performance.**^a^**

	Treatment^b^			
Item	Control	DFM	SEM	*P*-value
**BW, kg**				
**d 0**	26.48	26.98	0.346	0.33
**d 63**	43.31	43.05	0.589	0.77
**Experimental period (d 0 to 63)**				
**ADG, kg/d**	0.27	0.26	0.009	0.35
**ADFI, kg/d**	1.39	1.38	0.030	0.79
**G: F**	0.19	0.19	0.004	0.20

a A total of 72 lambs (Dorper × Katahdin; initially 26.73 ± 0.35 kg BW) were used in a 63-d growth trial with 3 lambs per pen and 12 replicate pens per treatment.

b Lambs were randomly assigned to either a control (no DFM supplementation) or a DFM (supplementation with 15 mL per day of RSG Prime by BIO S.I.).

Despite variable responses to DFM supplementation reported in the literature, several mechanisms have been proposed to explain potential changes in growth performance, including modulation of rumen microbial communities, stabilization of rumen pH, and enhanced fiber digestion (Newbold et al. 1996; McAllister et al. 2011). Certain strains of direct-fed microorganisms are known to promote the activity of lactate-utilizing and cellulolytic bacteria, which can shift rumen fermentation toward increased volatile fatty acid production, particularly propionate, thereby improving energy availability to the host (Oba and Allen 2003; Nagaraja and Titgemeyer 2007). However, these responses are often inconsistent, as they may depend on factors such as diet, animal age, microbial strain, and environmental conditions. Antwi et al. (2019) reported supplementation of DFM products to lambs did not influence growth performance. Additionally, Zulkifli et al. (2000) indicated temperature may cause inconsistent growth results for DFM-supplemented ruminants. The absence of treatment effects on growth parameters in the present study may reflect that lambs were already performing near their genetic capacity. Potential benefits may have occurred at the level of digestive function or microbial balance without translating to measurable improvements in BW gain or feed utilization within the 63-d period. Further research evaluating a longer post-supplementation period is warranted to determine whether this response represents a lasting advantage.

### Serum metabolites

There was no evidence of a significant treatment × day interaction for any of the measured serum metabolites (*p* > 0.05, Table 3), indicating that changes over time were independent of treatment. There was a tendency for blood calcium levels to be lower in lambs receiving the DFM supplementation compared to lambs in the control group (*p* = 0.06), suggesting a possible but inconclusive effect in nutrient metabolism and gut absorption that may warrant further investigation in larger studies.

**Table 3:** Effect of a liquid direct-fed microbial (DFM) on feedlot lamb blood serum chemistry.^a^

	Treatment			*P*-values		
Item	Control	DFM	SEM	Treatment	Day	Treatment × Day
***Albumin, g/dL***			0.027	0.78	< 0.01	0.30
*** d 0***	1.22	1.23				
*** d 21***	1.14	1.11				
***Glucose, mg/dL***			3.086	0.52	0.03	0.15
*** d 0***	76.50	79.50				
*** d 21***	88.83	82.08				
***Urea nitrogen, mg/dL***			1.294	0.95	0.02	0.63
*** d 0***	16.17	16.67				
*** d 21***	19.75	19.08				
***Calcium, mg/dL***			0.139	0.06	0.87	0.15
*** d 0***	10.46	10.43				
*** d 21***	10.71	10.23				
***Globulin, g/dL***			0.132	0.29	< 0.01	0.96
*** d 0***	2.74	2.93				
*** d 21***	2.96	3.15				
***Magnesium, mg/dL***			0.034	0.56	< 0.01	0.95
*** d 0***	0.84	0.87				
*** d 21***	0.95	0.97				
***Potassium, mmol/L***			0.025	0.10	0.06	0.44
*** d 0***	1.81	1.78				
*** d 21***	1.78	1.72				
***Bicarbonate, mmol/L***			0.065	0.91	< 0.01	0.69
*** d 0***	2.91	2.92				
*** d 21***	3.21	3.18				
***Anion gap, mmol/L***			0.047	0.84	< 0.01	0.94
*** d 0***	3.39	3.38				
*** d 21***	3.11	3.10				

a A whole blood sample was taken from one lamb per pen closest to the treatment average BW on d 0 and d 21. The same animal was sampled at both time points.

Several blood chemistry parameters demonstrated significant changes over time, regardless of treatment. Specifically, concentrations of potassium tended to decline (*p* = 0.06), while albumin and anion gap significantly decreased from d 0 to d 21 (*p* < 0.05). potentially reflecting physiological adaptations during the early feedlot transition period. The decline in serum albumin is consistent with the stress response observed during early feedlot transition (Tóthová et al. 2013). Potassium typically decreases extracellularly due to acidosis or a reduction in feed intake, however since no reduction in feed intake was observed, the decrease could be attributed to a decreasing rumen pH or intracellular shifts supporting tissue growth (Schneider et al. 2016). The reduction in anion gap may indicate stabilization of volatile fatty acid production and utilization as lambs transitioned to a new diet (Salles et al. 2012).

Conversely, levels of glucose, urea nitrogen, globulin, magnesium, and bicarbonate increased significantly from d 0 to d 21 (*p* < 0.05), likely reflecting metabolic adjustments associated with early growth and dietary adaptation. Glucose elevations are expected as lambs transition from forage to a concentrate-based diet, increasing energy availability. Additionally, this high-concentrate diet altered nitrogen metabolism as the lambs utilized different protein sources and levels when changing diets and continued to elevate as the rumen microbes adapted (Jin et al. 2018). Nitrogen cycling patterns change as the microbial populations establish in response to new substrates, resulting in increased urea production initially (Stewart and Smith 2005). Globulin production is a direct indicator of immune system activity over a stressful period. It has been shown that animals transitioning to feedlot conditions develop higher globulin levels due to exposure to diverse microbial challenges (Chovanová et al. 2021). The immune activation is a normal adaptive response that typically stabilizes once animals fully acclimate to their new environment. Magnesium concentrations similar to calcium are affected by blood pH and can act as an alkalizer in acidic conditions (Russel and Roussel, 2007). Serum electrolytes sodium, potassium, and chloride interact with bicarbonate to maintain acid-base equilibrium, with alterations in these parameters indicating shifts towards acidosis or alkalosis. The observed changes in magnesium and bicarbonate concentrations as lambs adapted to a new diet likely reflect metabolic adjustments and ruminal adaptation to a high-concentrate diet.

These results did not indicate that DFM supplementation facilitates metabolic efficiency during the critical transition phase following weaning. In addition to this, a significant day effect was noted in several blood biochemical parameters, irrespective of treatment. The observed temporal changes in blood metabolites highlight the complex interplay between dietary supplementation, ruminal health, and systemic physiological responses. Importantly, the lack of significant treatment-by-sampling day interactions suggests that both DFM and control groups experienced similar adaptive trends, possibly driven more by the natural course of post-weaning development than by the DFM intervention alone.

### Lamb fecal microbiota

Alpha diversity metrics including observed features (Amplicon Sequence Variants) and Faith’s Phylogenetic Diversity (Faith PD) were compared among treatment groups using Kruskal-Wallis pairwise tests. Observed features represent raw richness, defined as the number of unique sequences detected in a sample. Shannon diversity incorporates both richness and evenness, reflecting the number of taxa present and the distribution of their relative abundances. Faith PD accounts for phylogenetic relatedness and can distinguish communities with comparable richness but differing evolutionary diversity.

Pairwise Kruskal-Wallis comparisons for observed features identified significance in 4 instances. There was a time significance in both treatment groups with day 0 being significantly higher in observed features of alpha diversity than day 21 (*p* < 0.005; Table 4). Additionally, alpha diversity and microbial evenness in the control group on day 0 was significantly higher than the treatment group on day 21 (*p* < 0.007). Similarly, the treatment group on day 0 was significantly higher than the control group on day 21 (*p* ≤ 0.001). No significant differences were detected among groups for Shannon diversity (*p* ≥ 0.24). Faith PD demonstrated a pattern of significant differences comparable to those observed for the observed features metric.

**Table 4:** Pairwise Kriskal-Wallis comparisons of observed features alpha diversity among treatment and sampling day groups.^a^

		Statistical Values^b^		
Comparison Group		H-value	*P*-value	q-value
***Control d 0***	Control d 21	8.0033	0.0047	0.0093
***Control d 0***	DFM d 0	1.3333	0.2485	0.2979
***Control d 0***	DFM d 21	7.3633	0.0067	0.0100
***Control d 21***	DFM d 0	10.2720	0.0014	0.0041
***Control d 21***	DFM d 21	0.0033	0.9540	0.9540
***DFM d 0***	DFM d 21	11.0256	0.0009	0.0041

a On d 0 and 21, fresh fecal samples were collected from one lamb per pen closest to the treatment average BW (*n* = 12 lambs/treatment, 24 lambs total) for fecal microbiota analysis. Statistical comparisons of observed features were conducted using Kruskal-Wallis pairwise comparisons.

b H = Kruskal-Wallis test statistic; q = Benjamini-Hochberg false discovery rate-adjusted *P*-value.

**Figure 2:**
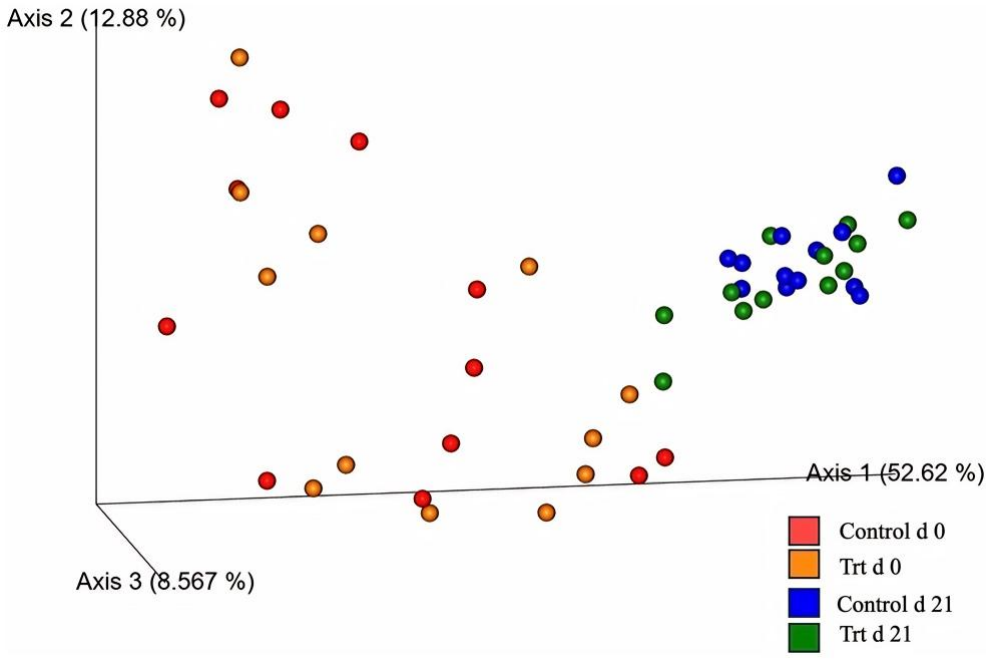
Principal coordinate plot of the microbial community structure between samples over time. On days 0 and 21, a fresh fecal sample was collected from one lamb per pen closest to the treatment average BW (*n* = 24) for analysis of fecal microbiota. Samples were stored at −80°C until DNA extraction and taxonomic analysis. Samples were analyzed using 16s rDNA sequencing. A ≥ 0.01% relative abundance threshold was set and samples not meeting this criterion were omitted from analysis. The microbial community structure, or Beta diversity, was analyzed using a weighted UniFrac distance matrix. A principal coordinate analysis plot was used to visualize the data in the distance matrix, and pairwise analysis of similarities (ANOSIM) was utilized to determine if there were any significant differences between the microbial communities. Samples collected on d 0, regardless of treatment, have a clear phylogenetic assemblage that differs significantly from samples collected on d 21 (*P* < 0.05). Primary vector explains 53% of the variation between the groups. The first 3 vectors together exhibit of 73% of the variation among the groups.

Beta diversity was assessed using weighted UniFrac distances to evaluate differences in microbial community structure among treatment and sampling day groups. Principal coordinate analysis (PCoA) was used to visualize patterns in community composition (Fig. 2). Pairwise analysis of similarity (ANOSIM) revealed significant differences in microbial community composition between day 0 and day 21 samples within both treatment groups (R ≥ 0.71, *p* ≤ 0.001; Table 5). In contrast, microbial communities did not differ between treatment groups within the same sampling day, as no significant differences were observed between control and DFM-treated lambs on day 0 (R = −0.05, *p* = 0.728) or day 21 (R = 0.10, *p* = 0.058). These results indicate that temporal changes in microbial community composition were more pronounced than treatment effects over the course of the study.

**Table 5:** Pairwise ANOSIM comparisons of microbial community composition among treatment and sampling day groups.^a^

		Statistical Values^b^		
Comparison Group		*R*	*P*-value	*q*-Value
***Control d 0***	Control d 21	0.7645	0.001	0.0015
***Control d 0***	DFM d 0	−0.0452	0.728	0.728
***Control d 0***	DFM d 21	0.7480	0.001	0.0015
***Control d 21***	DFM d 0	0.7442	0.001	0.0015
***Control d 21***	DFM d 21	0.0990	0.058	0.0696
***DFM d 0***	DFM d 21	0.7097	0.001	0.0015

a On d 0 and d 21, fresh fecal samples were collected from one lamb per pen closest to the treatment average BW (*n* = 12 lambs/treatment, 24 lambs total) for fecal micriobita analysis. Statistical comparisons of treatment and sampling day groups were conducted using the ANOSIM test.

b R = ANOSIM test statistic indicating the degree of separation between groups. A value closer to 1 indicates a greater dissimilarity; q = Benjamini-Hochberg false discovery rate-adjusted *P-*value.

Since lambs were allotted to treatments on d 0 and fecal samples collected for microbial analysis on d 0 were obtained prior to the initiation of DFM supplementation, the d 0 samples represent baseline microbial community composition before animals received the experimental treatment. While several taxa showed statistically significant differences on d 0 between treatment groups, these differences reflect natural inter-animal variability in the fecal microbiome rather than true treatment effects; thus, were not considered in the statistical model. Baseline variation in microbial community composition is to be expected as the microbiome develops differently amongst individual animals (Huang et al. 2022). Our primary objective was to investigate changes in the fecal microbiota over time, therefore the statistical model focused on post-treatment sampling days to determine whether DFM supplementation influenced microbial populations after treatment initiation. Fecal microbiota composition shifted over time, consistent with previous studies (Badman et al. 2019; Alawneh et al. 2024). It is difficult to conclude whether these differences directly reflect the influence of DFM supplementation on microbial community structure and function in conjunction with normal gastrointestinal development. At the Phyla level, the relative abundance of *Candidatus saccharibacteria* and *Verrucomicrobiota*, decreased from day 0 to 21 (*p* < 0.05, Table 6). *Verrucomicrobiota* exhibited a trend (*p* = 0.06) toward higher abundance in the DFM-treated group compared to control though, this was not statistically significant. At the class level, although *Opitutae* showed a statistically significant treatment x sampling day interaction (*p* = 0.03), the biological relevance of these differences is miniscule. Specifically, the relative abundance of *Opitutae* on d 0 was statistically different between treatments (*p* = 0.03); however, it is important to note that the numeric difference was not biologically relevant and the extremely low relative abundance across both treatments at both sampling days suggests that the d 0 differences likely are not attributed to treatment.

**Table 6:** Effect of a liquid direct-fed microbial (DFM) on the relative abundance of the most abundant taxa in feedlot lamb fecal samples.^a^

	Treatment^b^			*P*-values		
Item	Control	DFM	SEM	Treatment	Day	Treatment × Day
***Phyla***						
** *Verrucomicrobiota***			0.296	0.06	0.02	0.31
** d 0**	0.055	0.104				
** d 21**	0.004	0.038				
** *Candidatus saccharibacteria***			0.124	0.34	<.0001	0.34
** d 0**	0.005	0.002				
** d 21**	0.000	0.000				
***Class***						
** *Opitutae***				0.03	<0.001	0.03
** d 0**	0.004	0.001	0.112			
** d 21**	0.000	0.000				
***Order***						
** *Anaerolineales***			0.241	0.91	<.0001	0.91
** d 0**	0.013	0.011				
** d 21**	0.000	0.000				
** *Bifidobacteriales***			0.343	0.81	<.0001	0.04
** d 0**	4.390	20.090				
** d 21**	0.343	0.050				
** *Pirellulales***			0.168	0.64	<.0001	0.64
** d 0**	0.015	0.022				
** d 21**	0.000	0.000				
** *Kordiimonadales***			0.267	0.77	0.02	0.77
** d 0**	0.003	0.005				
** d 21**	0.000	0.000				
***Family***						
** *Francisellaceae***			0.211	0.71	0.01	0.64
** d 0**	0.004	0.004				
** d 21**	0.001	0.000				
** *Butyricicoccaceae***			0.247	0.90	<.0001	0.45
** d 0**	0.214	0.129				
** d 21**	0.008	0.012				
***Genus***						
** *Treponema***			0.314	0.54	0.02	0.03
** d 0**	0.285	2.310				
** d 21**	9.206	2.803				
** *Bifidobacterium***			0.348	0.87	<.0001	0.05
** d 0**	4.323	20.090				
** d 21**	0.302	0.049				

a A fresh fecal sample was collected from one lamb per pen closest to the treatment average BW on d 0 and d 21 (*n* = 24) for analysis of fecal microbial populations using 16 s DNA sequencing. The same lamb was sampled at both time points.

b Lambs were randomly assigned to either a control (no DFM supplementation) or a DFM (supplementation with 15 mL per day of RSG Prime by BIO S.I.).

Additionally, four specific orders; *Anaerolineales, Bifidobacteriales, Pirellulales*, and *Kordiimonadales*, showed significant decreases in relative abundance from day 0 to day 21 across both treatments (*p* < 0.02). This likely reflects a general adaptation to the environmental and dietary conditions of the study, as microbial communities stabilize over time. At the family level, *Francisellaceae* and *Butyricicoccaceae* also showed significant reductions over time (*p* < 0.05), reinforcing changes in microbial composition over time.

At the genus level, *Treponema* had a significant treatment × sampling day interaction (*p* = 0.03), whereas *Bifidobacterium* had a significant treatment × day interaction (*p* = 0.05). This interaction in *Bifidobacterium*, a genus often associated with gut health, may reflect competitive shifts in the microbial environment or dietary transitions.

Overall, these findings highlight that both time (acclimation) and treatment (DFM supplementation) contributed to shaping the fecal microbiota similar to previous studies (Fomenky et al. 2018; Campbell et al. 2024). Several taxa demonstrated treatment × sampling day interactions, suggesting that DFM supplementation my influence temporal shifts in the microbial community during transition periods. However, these interactions do not clearly indicate whether DFMs consistently promote stabilization of specific microbial groups. While some taxa exhibited patterns that could reflect responses to environmental or physiological stressors, the biological significance of these changes remains uncertain (Magalhães et al. 2024). Additionally, many genera from the Phyla, Order, and Genus level showed a significant change from day 0 to day 21, and *Opitutae*, *Bifidobacteriales*, and *Treponema* importantly showed a treatment x sampling day interaction. These patterns may reflect physiological adaptations associated with dietary and environmental transitions rather than a direct effect of a DFM supplementation (Cull et al. 2022). Despite these findings, several limitations should be acknowledged. Baseline microbial composition was not incorporated as a covariate in the statistical model, which may limit the ability to fully account for inherent inter-animal variability in the fecal microbiome. Additionally, the evaluation of microbial communities at only two time points restricts interpretation of the full temporal dynamics of microbial succession. Future work incorporating baseline-adjusted models and more frequent sampling would provide a more comprehensive understanding of how DFM supplementation influences microbial community development.

## Conclusions

In summary, supplementation with a DFM did not impact growth performance over a 63-d treatment period. All lambs, regardless of treatment, demonstrated changes in fecal consistency and blood biochemistry over time, which may provide insight into underlying physiological responses to diet and development. Fecal microbial populations differed between treatment groups and changed over time during the study period. While these patterns suggest that DFM supplementation may influence microbial community shifts during dietary and environmental transitions, the results do not clearly demonstrate that DFMs improved microbial adaptation during this period. Future studies should consider longer trial durations, alternative DFM strains or dosages, and more detailed assessments of rumen microbial dynamics to fully capture the potential benefits of DFM interventions in ruminant production systems.

## List of abbreviations

ADFI: Average daily feed intake
ADG: Average daily gain
ANOSIM: Analysis of similarities
BW: Body weight
CFU: Colony-forming unit
CON: Control (no supplementation)
DFM: Direct-fed microbial
Faith PD: Faith’s Phylogenetic Diversity
G: F: Gain-to-feed ratio
OTU: Operational taxonomic unit
PCoA: Principal coordinate analysis
P5P: pyridoxal 5’-phosphate
SPRI: Solid phase reversible immobilization
